# Optimization of Wavelength Dispersive X-Ray Spectrometry Analysis Conditions

**DOI:** 10.6028/jres.107.042

**Published:** 2002-12-01

**Authors:** Stephen J. B. Reed

**Affiliations:** Department of Earth Sciences, University of Cambridge, Cambridge CB2 3EQ, UK

**Keywords:** background, optimization, overlaps, virtual WDS, wavelength dispersive x-ray analysis, wavelength dispersive x-ray spectrometers

## Abstract

In setting up the conditions for quantitative wavelength-dispersive electron microprobe analysis a number of parameters have to be defined for each element, namely accelerating voltage, beam current, and (for each element) x-ray line, spectrometer crystal, pulse-height analyser settings, background offsets, and counting times for peak and background. The choices made affect both the reliability of the results and the time taken to obtain a complete analysis. It is difficult for even an experienced user to arrive at the optimum set of conditions for any particular application, in view of the large number of interacting factors involved. Furthermore, optimum choices of some parameters are dependent not only on the concentration of the element concerned (for example, counting times) but also the concentrations of other elements which may have peaks that interfere with peak and/or background measurements, requiring alternative selections of x-ray line or spectrometer crystal. The various factors involved in arriving at an optimum routine and practical possibilities for computer-aided optimization are discussed here.

## 1. Introduction

In quantitative electron probe microanalysis with wavelength-dispersive (WD) spectrometers, the operator is faced with a wide range of options in setting up an analysis routine. Obtaining reliable results in an efficient manner has traditionally depended almost entirely on the expertise of the operator but, with suitable software, the computer used to control the instrument and calculate the results can also be employed to assist in optimizing the analysis conditions. This can improve the quality of the results for the less experienced user and, in complex cases, even for an experienced one. The various factors involved in arriving at an optimum routine and practical possibilities for computer-aided optimization are discussed here.

## 2. Criteria for Optimization

The principal random errors in quantitative EPMA arise from statistical fluctuations in measured x-ray intensities, compared to which other sources of error, such as beam current instability, are usually negligible. For evaluating different options, the expression *n*^−1/2^ for the statistical (type A) relative uncertainty of one standard deviation, where *n* is the number of counts accumulated in the measurement of an x-ray peak intensity, can be used to evaluate either the counting time required to obtain a given uncertainty or the uncertainty obtainable for a given counting time, assuming the count-rate is known.

For major elements (where the peak intensity is much greater than background), the choice of x-ray line and of spectrometer crystal (where different choices are available) is governed simply by the criterion of maximum intensity (and hence minimum counting time for given statistical uncertainty). When the intensity measured at the peak position differs from background by only a small amount, the statistical uncertainty is governed by the expression *PB*^−1/2^ where *P* is the peak count-rate for the pure element, and *B* is the background count-rate on the sample. Peak-to-background ratio must then be taken into consideration as well as peak intensity in comparing options, which may lead to a different choice of conditions compared to those appropriate for a major element.

In some cases the first choice of x-ray line and crystal may not be the best, because of other considerations. For example, the existence of an overlapping line may lead to an alternative choice of either crystal (if this gives better resolution) or x-ray line, since if the intensity loss entailed is too great it may be preferable to tolerate the overlap and apply a correction for it.

## 3. Choice of X-Ray Line

For elements of atomic number less than approximately 30, the Kα line is nearly always the best (or only) choice. (The Lα line offers the advantage of higher spatial resolution obtainable with the low accelerating voltage permitted by the lower excitation energy, but is inappropriate for quantitative analysis for *Z* < 30, owing to uncertainties in the absorption correction.)

For *Z* > 30 the Lα line is a viable alternative to the Kα line. The higher overvoltage ratio (electron accelerating voltage divided by x-ray excitation energy) is a favorable factor as far as intensity is concerned but is counteracted by lower fluorescence yield. In addition, the observed intensities are significantly affected by spectrometer efficiency. In considering the optimum choice for low concentrations, peak-to-background ratio also comes into play (Sec. 2). The choice is thus not immediately obvious.

Certain criteria limiting the choice of x-ray line may be defined, though these are to some extent open to debate. It is undesirable for the overvoltage ratio to be less than 2, on the grounds that below this value intensity and peak-to-background ratio decline significantly. If one applies an upper accelerating voltage limit of 30 kV, then the Kα line ceases to be an option for *Z* > 36. For higher atomic numbers, the Lα line is the automatic first choice until the Mα line becomes available at *Z* = 70 (for lower atomic numbers the Mα line suffers from anomalous absorption and cannot be used for quantitative analysis). For higher atomic numbers, both Lα and Mα lines are available, though for Th and U the Lα line is excluded if the same criterion as that specified above for Kα lines is applied.

So far only lines α have been considered, on the grounds that they are nearly always more intense than β (or other) lines for a given element and shell. The main reason for choosing a less intense line is to avoid overlaps affecting the α line (Sec. 8). The loss of intensity by a factor of the order of 10 in the case of Kβ is, however, a considerable deterrent. (For Lβ and Mβ lines, the corresponding factor of around 2 is more acceptable.)

## 3. Choice of Spectrometer Crystal

**Fig 1:**
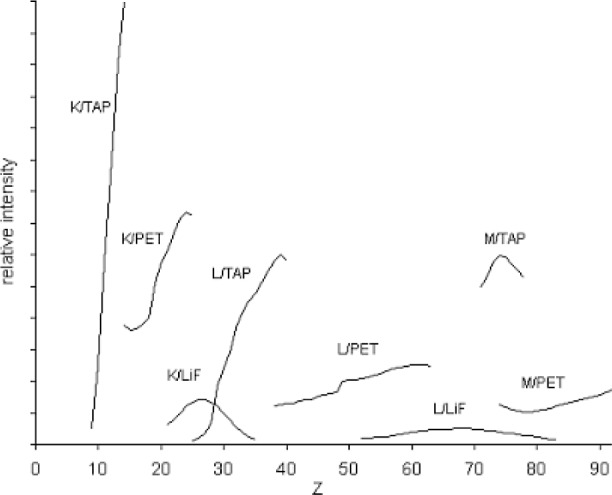
Typical relative intensities (in arbitrary units) of K, L, and M α lines of pure elements as a function of atomic number (*Z*), for 20 kV accelerating voltage, using TAP, PET, and LiF spectrometer crystals. Where the wavelength ranges overlap, the crystal with the larger interplanar spacing gives the higher intensity, but sometimes the presence of interferences necessitates the use of the crystal with smaller spacing, on account of the higher wavelength resolution obtained. The wide range of intensities must be taken into account in selecting optimal counting times.

The wavelength ranges covered by the crystals used in WD spectrometers (as governed by the available Bragg angle range for the spectrometer design concerned) overlap to a certain extent (more between PET and LiF than between TAP and PET). For wavelengths lying within the overlapping regions, choice of crystal is thus a further consideration. This choice is linked with that of x-ray line, since observed intensities are affected both by generated x-ray intensity and by spectrometer efficiency (see Fig. 1).

The efficiency of WD spectrometers is dependent on various factors including (a) the solid angle subtended by the crystal, (b) the reflection efficiency of the crystal, and (c) the detection efficiency of the proportional counter. Both (a) and (b) show a predominantly downward trend with increasing Bragg angle, *θ* [1,2]. At the same time the resolution, defined as λ/Δλ (where λ is the wavelength and Δλ the peak width), increases with *θ*, owing to the decreasing effect of aberrations of the curved crystal geometry, though this is subject to modification owing to variations in counter efficiency. It follows that where a choice of crystal for a given x-ray line is available, the one with larger interplanar spacing (and hence lower Bragg angle) gives higher intensity. However, the peak-to-background ratio is greater for the crystal of smaller spacing, owing to the better resolution and consequently narrower band of x-ray continuum detected, which may lead to the opposite choice being preferable. This also applies to cases of overlapping lines, where resolution is important (Sec. 8).

Manufacturers of electron microprobe instruments usually offer a choice of crystal size. Selecting the larger size may restrict the number of crystals that can be accommodated per spectrometer (2 instead of 4, for example), which limits the possibility of using the same type of crystal in more than one spectrometer when a large proportion of the relevant x-ray lines lie within the range of that crystal (though with the usual full complement of five spectrometers this is rarely a serious issue).

Increasing the crystal size increases the reflected intensity but for geometrical reasons there is an associated sacrifice in resolution and hence peak-to-background ratio, though less so if Johansson geometry (crystal curved and ground) is used in place of Johann geometry (crystal curved but not ground) [1,2,3], though fabrication is more difficult. For low concentrations, some of the advantage conferred by the gain in intensity is therefore counteracted by the decrease in peak to background ratio.

## 4. Accelerating Voltage

The electron accelerating voltage must be high enough to excite all the relevant X-ray lines, preferably with an overvoltage ratio of at least 2. The generated intensities of x-ray lines increase with increasing accelerating voltage, but for heavily absorbed lines (notably those of long wavelength) the effect of self-absorption can dominate above a certain voltage, causing the intensity to decrease. Even before this stage is reached, the size of the absorption correction may significantly degrade the accuracy of quantitative analysis. These considerations determine the optimum accelerating voltage for a given element, while for multi-element analyses a compromise in necessary.

The continuum intensity also increases with accelerating voltage, but less rapidly, so that the peak-to-background ratio increases, which is favourable for elements present in low concentrations. For minor and trace elements, a higher voltage is often desirable than is optimal for major elements, where the accuracy of the absorption correction is more important (this being less crucial for trace elements, for which statistical uncertainty is the limiting factor). A compromise choice is therefore required, depending on relative priorities. Alternatively, the analysis may be carried out in two separate stages optimised for major and trace elements respectively [4,5].

The effect of the choice of accelerating voltage on spatial resolution may also require consideration. The need to limit the voltage in order to obtain a certain resolution necessitates longer counting times to compensate for the reduced intensities, and may also require the choice of x-ray lines of lower excitation energy (L rather than K, or M rather than L).

## 5. Beam Current

A high beam current is advantageous owing to the proportionate effect this has on x-ray intensities, allowing shorter counting times for a given statistical uncertainty, or smaller uncertainty for given times. In some cases the increase in beam diameter which occurs as the condenser lens settings are changed may dictate an upper current limit. Another consideration is overloading of the counting system for intense peaks, resulting in pulse height depression and possible errors in dead-time corrections. In cases where a very high current is desirable for trace element measurements, the two-stage procedure described above may be adopted. An alternative possibility is to use an energy-dispersive (ED) detector with a small entrance aperture for major elements.

For some types of sample, damage by the electron beam may affect quantitative analysis results. The usual strategies for minimizing damage, such as rastering the beam, increasing the beam diameter, or coating the sample with a thicker than usual conducting layer, are reasonably effective, but an upper limit to the current may still be desirable. The accelerating voltage is also relevant in this context. It is commonly assumed that a high value increases the probability of damage. However, this is often not so, since, for a given current, the energy dissipated per unit volume in the sample is less for a high voltage because, although the total energy deposited in the sample increases with voltage, the volume penetrated increases much more rapidly.

## 6. Background Measurement

Characteristic x-ray lines from the elements in the sample are superimposed on the x-ray continuum, and the intensity measured with the spectrometer set on the peak requires correction for the contribution of the continuum background underlying the peak. The background correction is usually determined by measuring the intensity with the spectrometer offset by a suitable amount on each side of the peak and interpolating linearly to the peak position. The counting times used for background can be quite short for major peaks, for which the peak-to-background ratio is large, and the choice of time is not critical. For very small peaks the lowest statistical uncertainty for a given total counting time is obtained if the time used for measuring background is the same as for the peak. (It is a simple matter to calculate the optimal division of time in the general case, though this requires prior knowledge of the relevant intensities.)

The intensity of the continuum background varies sufficiently slowly as a function of wavelength that linear interpolation is accurate enough except possibly for very low concentrations, long counting times, high beam current, etc. The effect of background curvature is more likely to be significant for samples of high mean atomic number, since the continuum intensity is then greatest, and a given degree of curvature has a greater effect in terms of elemental concentration. The effect of curvature can be minimised by using the smallest practicable background offsets. If necessary a curvature factor can be determined from a material known to contain none of the relevant element.

For major elements the background is often so small that a simplified form of correction is permissible, thereby saving the time required for the usual offset measurement. Background intensities measured in previous analyses or derived from modelling calculations can be utilised, but should be scaled according to mean atomic number to allow for the effect this has on continuum intensity.

## 7. Overlaps

Line overlaps affecting elemental peaks can be corrected by subtracting the contribution of the overlapping peak, but nevertheless should be avoided if possible, in view of the uncertainties in the correction, and increased statistical uncertainty resulting from the increased effective background. In optimizing analytical conditions, a procedure is therefore required for predicting the presence of overlaps and considering alternative choices of x-ray line (for example, β instead of α, or M instead of L, etc.), and/or spectrometer crystal (one with a smaller interplanar spacing, giving higher resolution). Wavelength tables are of rather limited usefulness in this context because it is difficult to evaluate the influence of a nearby line as a function of its distance from the peak of interest; also, the heights of the peaks concerned need to be taken into account. The need for time-consuming spectrometer scans to investigate overlaps for each application can be obviated by making use of simulated spectra, as described in the following section.

## 8. Spectrum Simulation

It has been found useful to simulate energy-dispersive spectra as an aid to ED analysis, as in the NIST/NIH Desk Top Spectrum Analyzer (DTSA) program. Problems entailed in adapting this to WD spectra are discussed in Ref. [6]. Peak shapes vary within the range of a given crystal, from being asymmetric and more rounded in shape at low Bragg angles to being symmetric and more pointed at high angles, requiring a varying proportion of gaussian and lorentzian functions in the “pseudo-Voigt” peak shape function [7]. Spectrometer efficiency also varies strongly as a function of Bragg angle (see Sec. 3) and, in the absence of first-principles calculation methods, has to be represented by empirical curves [1]. A further problem with spectrum synthesis is the inadequacy of available tabulated data on x-ray line intensities, especially for minor lines and long wavelengths.

**Fig. 2:**
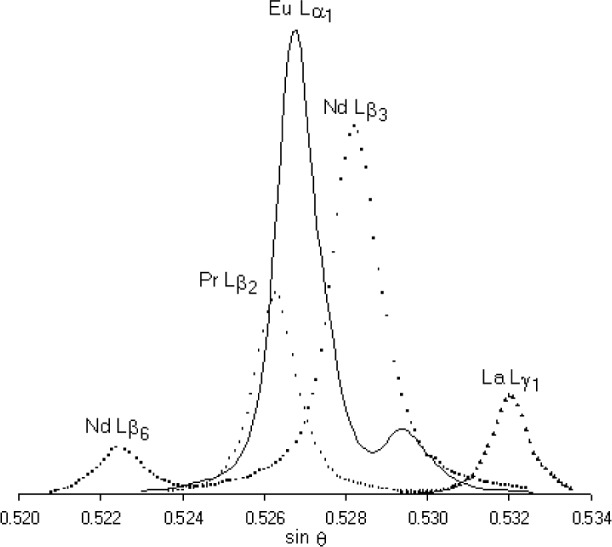
Simulated spectra of rare-earth elements (LiF crystal), with relative concentrations equal to average values for the Earth’s crust (LiF crystal). The Eu Lα_1_ peak suffers interference from peaks of other REE, which invariably coexist in natural REE-bearing minerals. In such phases the relative REE abundances commonly show enrichment or depletion of light relative to heavy REE, the implications of which for WD analysis may be studied by spectrum simulation [Ref. 9]. Optimum background offsets can be selected without the need for time-consuming spectral plots on actual specimens.

These difficulties are avoided in the “Virtual WDS” program [8], which contains a complete database of experimental spectra from which a visual display of a given elemental peak and any potentially interfering peaks of other elements is generated. This can be used to reveal the presence and size of overlaps from the peaks of any elements, including peaks arising from high-order reflections. If the sample composition is known at least approximately, more specific information can be obtained by including only relevant peaks and scaling these according to concentration. Spectrum simulation is an important tool for quantitative WD analysis, especially for elements in low concentrations, because failure to recognise overlaps can lead to serious analytical errors. (For an example of an application, see Fig. 2).

This approach can also be applied to background measurements, which are just as significant since overlaps lead to the under-estimation of the concentration, and even negative or “false zero” values. Such effects can often be minimised by optimal choice of the points at which backgrounds are measured. Also, in some cases a single measurement on one side of the peak only may be the best option. Where it is impossible to eliminate overlap effects completely, spectrum simulation may be applied to the estimation of the correction required as a function of the concentration of the overlapping element.

## 9. Counting Times

Having made selections for the various parameters discussed above, it remains to decide on the counting times to use. Since a major objective of optimization is to achieve a certain level of uncertainty for all elements in the least possible time, counting times should be no longer than are required to obtain the target statistical uncertainty (Sec. 2). Little is gained by aiming for better than ± 1 %, since other factors generally limit overall uncertainty to around this figure. To achieve optimal efficiency the wide variation in count-rate between different elements, lines and crystals (see Fig. 1) should be taken into account. Also, the element concentrations need to be known at least approximately, and the times adjusted accordingly (unless the “fixed count” mode is used, whereby counting continues until a suitable total is reached). Optimizing counting times is, of course, much more critical for low concentrations, for which the times are long, than for major elements.

## 10. Analysis Strategy

Traditionally, the microprobe operator is presented with a wide range of choices in setting up an analytical routine, which places considerable demands upon his or her expertise and ability to manipulate simultaneously all the various considerations covered in the preceding sections. It is clearly desirable that the computer used to control the instrument and calculate the results should be applied to this problem.

Various levels of assistance to the operator are possible. The simplest is the provision of “defaults” for all parameters, instead of blank spaces. These should be reasonable choices for a wide range of applications (or possibly stored values saved from previous similar applications), and reduce the possibility of inexperienced operators making bad choices. The conditions can be manually edited to make them better suited to particular applications. This is facilitated by using a software package such as “Virtual WDS” [8], into which information on the elements present and, if possible, their approximate concentrations, can be entered so that choices of x-ray lines, spectrometer crystals, and background offsets can be revised to avoid overlaps. Also, information on count-rates contained in the database can be utilised to determine counting times required for given statistical precision.

In the “expert system” developed by Fournier et al. [10], the user provides information on the number and configuration of the spectrometers (types of crystal and counter), estimated specimen composition (if known), required precision for all elements present (or time available for complete analysis), spatial resolution required, and maximum permissible beam current. A set of analysis configurations is created, consisting of all possible combinations of x-ray lines and crystals that satisfy certain criteria. The lines considered initially are those K, L, or M α lines that are adequately excited (see Sec. 3), and each of these is linked with one or more spectrometer crystal. If interferences exceeding 5 % of the peak intensity are predicted by a spectrum simulation model, then the β line is substituted (a warning is given if no interference-free line/crystal combinations for a given element exist). This process is repeated for different accelerating voltages. For each of these, generated x-ray intensities are calculated and combined with empirical spectrometer efficiency data to give predicted countrates. Counting times are then selected to give the specified statistical uncertainty (see Sec. 2) and the configuration which gives the minimum total time per analysis (allowing for spectrometer movement between peaks) is determined. The beam current is set to the maximum permissible, consistent with count-rates that do not exceed the limit beyond which overload effects occur (see Sec. 5). The optimum accelerating voltage is determined by comparing the chosen configuration for each voltage value, taking account of any spatial resolution requirement specified by the user, which may impose an upper limit. In evaluating uncertainty, that attributable to the absorption correction is added quadratically to the statistical counting uncertainty, so that the increasing absorption errors are taken into account.

## 11. Standardless WD Analysis

Prior knowledge of the sample composition (at least approximately) is a key requirement in any scheme for optimising WD analysis conditions. Sometimes this information is already available, but otherwise some form of preliminary analysis is necessary. For this purpose ED analysis may be adequate. In the “standardless” mode, calculated intensities are combined with a model of detection efficiency that takes account of window absorption and other factors (see, for example, Ref. [11]). This has the advantage that no assumption about which elements are present is required and standard calibration is unnecessary. Standardless ED analysis has unavoidable limitations when applied to spectra with seriously overlapping peaks, but the large uncertainty sometimes attributed to this method [12] is due partly to inadequacies in the data used for intensity calculations, which undoubtedly could be improved.

A preliminary WD analysis (with better resolution and peak to background ratio) is a possible alternative. Using the conventional approach, prior knowledge of the elements present is required, but this can be circumvented by using a procedure in which the whole x-ray spectrum is covered by rapid scanning. In the method described in Ref. [13] each spectrometer scan is represented by about 1000 data points, with a total recording time of 2 min. After identifying all statistically significant peaks, intensities are obtained by fitting a peak profile function, and semi-quantitative (± 10 % approximately) concentrations are derived from the intensities of the principal lines (usually α lines, unless overlaps require an alternative choice) using either calculated elemental intensities or previously recorded experimental data.

Scanning WD spectrometers is a rather inefficient mode of data collection, owing to the narrowness of the peaks and the large fraction of the spectrum containing only background. A possible alternative is to record only regions containing major x-ray lines. It is desirable to apply overlap corrections where necessary, for which predetermined factors can be used [14]. Some allowance must be made for uncertainty in the exact positions of peaks, which in conventional quantitative WD analysis are determined by peak-seeking on standards (though rapid standardless analysis inherently has larger uncertainty and is therefore less demanding in this respect).
